# Experience of Endoscopic Dilatation of Esophageal Strictures in a Tertiary Care Hospital: A Study From Central India

**DOI:** 10.7759/cureus.76545

**Published:** 2024-12-28

**Authors:** Mohd Talha Noor, Shreyansh Talera, Rootik Raju Bhai Patel, Nidhesh Khemchandani

**Affiliations:** 1 Department of Gastroenterology and Hepatology, Sri Aurobindo Medical College and Post Graduate Institute, Indore, IND

**Keywords:** endoscopic dilatation, esophageal strictures, post-corrosive strictures, post radiation, savary-gilliard dilatation

## Abstract

Background

Endoscopic dilatation is the cornerstone therapy for esophageal strictures. The primary indication for dilatation is to provide immediate and durable symptomatic relief from dysphagia. Following esophageal dilatation, the two most common major consequences are bleeding and perforation, both of which are quite rare. Post-radiation and caustic strictures continue to be a common source of recurrent and refractory strictures that require multiple dilatations, despite a variety of therapeutic techniques. There is a lack of studies dealing with the efficacy and safety of the procedure. Therefore, this study was conducted to explore the effectiveness and application of the Savary-Gilliard dilatation of strictures, as well as the factors influencing the outcome.

Methods

We recruited patients of dysphagia of various grades having esophageal strictures of various etiologies. The dilatation sessions were done as an outpatient procedure in the majority of patients. In all patients, preprocedural workups, such as routine investigations and esophagogastroduodenoscopy, were done. Contrast-enhanced computed tomography scans of the abdomen and barium swallow examination were done in selected patients. Results of dilatation using wire-guided dilators were reported as the number of dilatations per stricture according to their location and the mean number of sessions required to achieve the target diameter (15 mm). Evaluating clinical efficacy and adverse events were the main goals.

Results

A total of 250 dilatations were carried out in 112 patients, of whom 67 were females and 45 were males. The mean age of the study population was 50.38 ± 15.4 years. Post-radiation stricture was the commonest. Strictures of upper esophageal origin required the highest number of sessions (2.21 ± 0.78) for adequate dilatation compared to the middle (2.14 ± 0.83) and lower locations (1.7 ± 0.78) with a p-value of 0.004. Post-corrosive upper esophageal stricture required a greater number of sessions for adequate dilatation as compared to post-radiation strictures (3.0 ± 0 vs. 2.6 ± 0.63) with a p-value of 0.005.

Conclusion

Esophageal dilation for strictures produces a good outcome with minimal adverse effects or major complications. Post-radiation, post-surgical, and post-corrosive strictures are more likely to require repeated sessions for symptomatic relief.

## Introduction

Patients with esophageal strictures initially present with dysphagia of various grades. The diameter of the esophageal lumen, less than 12 mm, with the extent of the reduction diameter of the esophageal lumen, is closely correlated with the severity of dysphagia [1,2]. The primary goal of dilatation is to treat dysphagia symptoms immediately and permanently. Currently, there are two kinds of esophageal dilators in use: Cook Medical's Savary-Gilliard wire-guided polyvinyl dilators and balloon dilators. The Savary-Gilliard dilation is safer than bougie dilation because it makes sure the dilator stays in the axis of the lumen and does not buckle or bend laterally into the stricture's wall, which would increase the risk of perforation. Endoscopic dilatation with bougies or balloons is an effective treatment for around 80-90% of esophageal stricture. Following esophageal dilatation, the two most common major consequences are bleeding and perforation [3,4,5]. Most of these individuals require repeated dilatations based on etiology and type of stricture [2,6].

Simple strictures are usually short (<2 cm) and straight and allow passage of diagnostic endoscopes. An example of a simple esophageal stricture is a Schatzki ring. Simple strictures are quite easy to manage [7]. In general, dysphagia due to simple strictures can be relieved with one to three dilatations. Only 25-35% of patients need follow-up appointments and over 95% of patients only need a maximum of five dilations [4]. Complex strictures are longer (>2 cm), tortuous, and angulated or have a severely narrowed diameter and often do not allow passage of an endoscope. Complex strictures can be mainly caused by surgery, radiotherapy, or corrosive [8]. In addition to being more challenging to treat, these strictures frequently become recalcitrant to dilation therapy or reoccur. Circular, anastomotic strictures are among the more common and complicated strictures, even when there is no endoscopic indication of inflammation [9].

Endoscopic dilatation is the cornerstone therapy for the management of esophageal strictures. The type of stricture greatly influences the therapeutic modality employed for dilatation [10]. A majority of patients have only partial blockage, which can be treated with balloon dilators inserted through the scope, with the use of guide-wire or with wire-guided rigid polyvinyl dilators [11]. Radiation strictures and caustic strictures continue to be leading causes of recurrent and refractory strictures in spite of these treatment options as pointed out by Lee J et al. [12].

A stricture is classified as refractory if it is unable to dilate to a 15 mm diameter after five or more sessions in 10 weeks. A recurrent stricture was defined as the recurrence of difficulty in deglutition and the inability to maintain a satisfactory luminal diameter for four weeks once the target diameter of 15 mm had been achieved. The inability to achieve clinical success was deemed a failure [13].

Although endoscopic dilatation is a commonly performed procedure, there is a lack of studies dealing with its efficacy and safety. Hence, The purpose of this study was to investigate the efficacy and safety of the Savary-Galliard dilatation of strictures and the various factors affecting the outcome.

## Materials and methods

This was a cross-sectional study performed at the Department of Gastroenterology of Sri Aurobindo Medical College and Post Graduate Institute, Indore, India, from January 2016 to December 2023 after obtaining approval from the Institutional Ethics Committee (approval no. SAIMS/RC/IEC/22/2). The dilatation sessions were done as an outpatient procedure in the majority of patients. In all patients, preprocedural workup was done such as routine investigations and esophagogastroduodenoscopy. Contrast-enhanced computed tomography scans of the abdomen and barium swallow examination were done in selected patients. Inclusion criteria were (1) symptomatic patients of esophageal stricture (dysphagia grade 2 or more) and (2) narrowing seen on endoscopy and barium swallow examination. Patients younger than 18 years of age or who had not given their consent to participate in the study were excluded from the study. Before dilation, written informed consent was obtained from each patient. Patients were sedated by anesthetic using intravenous midazolam (30 g/kg) prior to endoscopic dilatation. Endoscopic dilatation was performed by wire-guided bougie (Savary-Gilliard dilators, Wilson-Cook Medical Inc., Winston-Salem, NC). In each session, not more than three dilators were passed. Fluoroscopic guidance was used in selected cases. Every third week, dilatations were performed repeatedly until the target diameter was achieved. During the procedure, the initial dilator size was chosen by the operator on the basis of imaging findings and endoscopist subjective assessment. Patients with refractory strictures were considered for endoscopic injection of steroids into the stricture. Triamcinolone acetonide (80 mg, diluted 1:1 with a saline solution) was injected with a 23-gauge, 5-mm-long sclerotherapy needle (Olympus Optical Co., Tokyo, Japan) in aliquots of 0.5 mL each as described in detail elsewhere [13].

The strength of our study was that in selected cases, a pediatric scope was passed under fluoroscopic guidance to confirm the position of the guidewire. The endpoint of dilation was the clinical success to reach a target diameter of 15 mm. After each dilation, the patient was observed for four hours with specific attention to chest pain, difficulty in breathing, and hemodynamic status. Patients were discharged on the same day with instructions to immediately report in case of any complications. In the event of suspected perforation, a water-soluble contrast study was performed to witness any leak, and an urgent surgical consultation was taken. All patients were followed up with a clinical and endoscopic examination once every three months for six months. Adverse occurrences were classified as early or late based on when they happened within seven days and after seven days. For patients who were not able to come for follow up phone calls were made every six months to evaluate the alleviation of their symptoms.

Statistical analysis

The presentation of the categorical variables was done in the form of numbers and percentages (%). On the other hand, the quantitative data with normal distribution were presented as the means ± SD. The association of the variables, which were quantitative in nature, was analyzed using an independent t-test (for two groups) and an ANOVA test (for more than two groups). The association of the variables, which were qualitative in nature, was analyzed using the chi-square test. If any cell had an expected value of less than 5, then Fisher’s exact test was used. The data entry was done in the Microsoft Excel spreadsheet (Microsoft Corp., USA), and the final analysis was done with the use of IBM SPSS Statistics for Windows, version 25.0 (released 2017, IBM Corp., Armonk, NY). For statistical significance, a p-value of less than 0.05 was considered statistically significant.

## Results

Table 1 shows the patient characteristics. One hundred twelve patients received a total of 250 dilations, 45 of whom were men and 67 of whom were women. The study population's average age was 50.38 ± 15.4 years. The patients were followed up for a mean period of six months. Successful dilatation was achieved in 73.21% of patients (82 out of 112), and the mean number of sessions required to achieve adequate dilatation was 2.02 ± 0.8. Three patients (2.68%) had refractory strictures and three (2.68%) had recurrent strictures. Strictures of upper esophageal origin required the highest number of sessions (2.21 ± 0.78) for successful dilatation compared to the middle (2.14 ± 0.83) and lower locations (1.7 ± 0.78). A statistically significant difference was observed with a p-value of 0.004. The results are shown in Table 2. Strictures that were refractory to endoscopic dilatation were post-radiation and post-corrosive in nature. Recurrent strictures belonged to the post-radiation group, as shown in Table 3.

**Table 1:** Demographic characteristics distribution

Demographic characteristics	N(%)
Gender	
Female	67 (59.82%)
Male	45 (40.18%)
Weight loss	83 (74.11%)
Tobacco consumption	33 (29.46%)
Location	
Upper	45 (40.18%)
Middle	34 (30.36%)
Lower	33 (29.46%)
Aetiology	
Peptic stricture	17 (15.18%)
Post radiation	51 (45.54%)
Post corrosive	15 (13.39%)
Esophageal web	14 (12.5%)
Post-surgical	09 (08.04%)
Esophageal ring	03 (02.68%)
Others	03 (02.68%)

**Table 2:** Comparison of outcomes of endoscopic dilation with location using Fischer's exact test and ANOVA test

Variables	Lower (N = 33)	Middle (N = 34)	Upper (N = 45)	Total	P-value
Successful dilation	27 (81.82%)	22 (64.71%)	33 (73.33%)	82 (73.21%)	0.252
Recurrent	0 (0.00%)	3 (8.82%)	0 (0.00%)	3 (2.68%)	
Refractory	0 (0.00%)	1 (2.94%)	2 (4.44%)	3 (2.68%)	
Mean ± SD sessions required for successful dilation	1.7 ± 0.78	2.14 ± 0.83	2.21 ± 0.78	2.02 ± 0.82	0.004

**Table 3:** Distribution of outcomes in various etiologies.

Outcomes	Peptic stricture	Post radiation	Post corrosive	Esophageal web	Esophageal ring	Post surgical	Others
Successful dilatation	16 (94.12%)	33 (64.71%)	08 (53.33%)	14 (100%)	03 (100%)	06 (66.67%)	02 (66.67%)
Upper location	0	15 (45%)	1 (13%)	14 (100%)	0	3 (50%)	0
Middle location	4 (25%)	13 (40%)	2 (25%)	0	0	2 (33%)	1 (50%)
Lower location	12 (75%)	5 (15%)	5 (62%)	0	3 (100%)	1 (17%)	1 (50%)
Recurrent	0	3 (5.88%)	0	0	0	0	0
Refractory	0	2 (3.92%)	1 (6.67%)	0	0	0	0

Stricture etiology was post-radiation in 51 patients, peptic 17, post-corrosive 15, esophageal web 14, post-surgical nine, esophageal ring three, and other causes three patients (Table 1). The average (±standard deviation) number of sessions for successful dilatation needed was 1.81 ± 0.91 for peptic stricture, 2.39 ± 0.75 for post-radiation, 2.00 ± 0.76 for post-corrosive, 1.71 ± 0.61 for esophageal ring, 2.17 ± 0.75 for post-surgical, and 1.71 ± 0.6 for esophageal web. Post-radiation strictures required the highest number of sessions for adequate dilatation, followed by post-surgical and post-corrosive strictures, respectively (2.39 ± 0.75 vs. 2.17 ± 0.75 vs. 2.00 ±.76) with a significant p-value of 0.004. Post-corrosive upper esophageal stricture required a greater number of sessions for adequate dilatation as compared to post-radiation (3 ± 0 vs. 2.6 ± 0.63). This difference was statistically significant with a p-value of 0.005. The results are shown in Table 4.

**Table 4:** Comparison of number of sessions for successful dilatation in various locations and etiology as per ANOVA test. Data has been represented as mean ± SD and p-value <0.05 is considered significant.

Number of sessions for successful dilatation	Peptic stricture	Post radiation	Post corrosive	Esophageal web	Esophageal ring	Post surgical	Others	p-value
Upper location	-	2.6 ± 0.63	3 ± 0	1.71 ± 0.61	-	2.33 ± 1.15	-	0.005
Middle location	2 ± 1.41	2.31 ± 0.75	2 ± 0	-	-	2 ± 0	1 ± 0	0.663
Lower location	1.75 ± 0.75	2 ± 1	1.8 ± 0.84	-	1 ± 0	2 ± 0	1 ± 0	0.543
Mean number of sessions for successful dilatation for upper, middle, and lower locations (overall)	1.81 ± 0.91	2.39 ± 0.75	2 ± 0.76	1.71 ± 0.61	1 ± 0	2.17 ± 0.75	1 ± 0	0.004

## Discussion

In this study, we performed a total of 250 dilatations in 67 females and 45 males. The study population's mean age was 50.38 ± 15.4 years. Our results showed endoscopic dilatation is a useful technique for the management of dysphagia, with the overall clinical success achieved in 73.21% of patients and the mean number of dilatations required to achieve adequate dilation were 2.02 ± 0.8. Response to dilatation was better in patients with peptic strictures, esophageal webs, and esophageal rings than post-corrosive, post-radiation, and post-surgical strictures. Van Heijl et al. [14] found that 83% of their patients experienced clinical success with a median of five dilatation sessions. Mendelson et al. [15] found a high rate of success using frequent dilatation sessions (median 3).

The average (±standard deviation) number of sessions needed was 1.81 ± 0.91 for peptic stricture, 2.39 ± 0.75 for post-radiation, 2.00 ± 0.76 for post-corrosive, 1.71 ± 0.61 for esophageal ring, and 2.17 ± 0.75 for post-surgical. Post-radiation strictures required the highest number of sessions for adequate dilatation, followed by post-surgical and post-corrosive strictures (2.39 ± 0.75 vs. 2.17 ± 0.75 vs. 2.00 ± 0.76) with a significant p-value of 0.004. It was pointed out by Vermeulen et al. [16] that caustic strictures required the highest number of mean dilatations, followed by post-radiation strictures to attain sufficient dilatation, with 7.2 (±2.2) in caustic strictures, 5.0 (±2.6) in radiation, 4.9 (±2.3) in anastomotic, 3.6 (±2.4) in peptic, 3.8 (±2.7) in post-endo therapy, and 1.8 (±1.9) in Schatzki rings.

In our study, three patients had a refractory stricture of post-radiation and post-corrosive etiology, while three patients had a recurrent stricture of post-radiation etiology. Furthermore, we observed that, in comparison to the middle (2.14 ± 0.83) and lower locations (1.7 ± 0.78), strictures of the upper esophageal origin required the greatest number of sessions (2.21 ± 0.78) for sufficient dilatation. This difference was determined to be statistically significant, with a p-value of 0.004. While a handful of studies have linked their results to the stricture etiology, none have examined how many sessions are needed depending on the strictures' anatomical position. Our study is unique in having an association of stricture etiology with strictures on anatomical position.

As per the location, our center had the highest number of cases of post-radiation strictures (15), followed by esophageal web (14) post-surgery (3), and post-corrosive (1) patients in the upper location. The mean sessions required for adequate dilation were maximum in post-corrosive patients (3 ± 0), followed by post-radiation (2.6 ± 0.63) and post-surgical (2.33 ± 1.15). Esophageal web patients required less dilation (1.71 ± 0.61) as compared to other etiologies in the upper location with a significant p-value = 0.005.

The middle location had the highest cases of post-radiation strictures (13), followed by peptic strictures (4). Post-radiation strictures required maximum dilation (2.31 ± 0.75) in the middle location, followed by post-corrosive (2.0 ± 0) and peptic strictures (2.0 ± 1.41).

The lower location had the highest number of peptic strictures (12), followed by post-radiation (5) and post-corrosive (5) strictures. The mean number of sessions required for adequate dilation was maximum in post-radiation (2 ± 1), followed by post-corrosive (1.8 ± 0.84) and peptic strictures (1.75 ± 0.75).

The efficacy of dilatations in benign strictures was dependent upon the degree of fibrosis and inflammation that had affected the esophageal wall. Caustic, postoperative, and post-radiation therapy strictures are the groups that respond the least to dilatation. This was consistent with the information provided in other publications [17].

The entire thickness of the esophageal wall is involved and the strictures are known to extend in length. As a result, the damaged esophagus tubulates, making it harder to dilate effectively. Leuba et al. presented the caustic-stricture dilatations' results [18]. They discovered that if the stenosis extends beyond 3 cm In the cranio-caudal axis, dilatations are often inadequate for the management of dysphagia. If symptoms do not improve after a few dilatation sessions, an esophagectomy may be necessary to restore normal oral intake.

Healing in patients who have consumed caustic substances involves collagen deposition and fibrosis, resulting in cicatrization of varying lengths of the esophagus adjacent to the stricture. This process can result in the creation of recalcitrant strictures in such patients. Esophageal strictures due to caustic acid led to long-lasting swallowing difficulties, which were attributed to the cicatrization of the surrounding esophageal tissue and resultant motor dysfunction. Ingestion of caustic substances is the main cause of oesophageal strictures in young children and adolescents [19].

Murphy et al. pointed out that radiotherapy is an essential treatment for cancers of the esophagus, head, neck, and chest. Numerous conditions can cause dysphagia including radiation dermatitis, xerostomia, mucositis, and edema in the acute phase. Later, it can cause fibrosis, neuromuscular damage, lymphedema, and mechanical blockage [20]. The treatment of head and neck, lung, esophageal, and breast cancers frequently results in late radiation damage. Sluggishly developing fibrosis, ischemia, and gradual obliterative endarteritis are associated with the development of strictures [21].

The total absorbed dose is the main factor that predisposes a patient to developing strictures after radiation therapy. Patients are more likely to develop strictures at doses higher than 60 Gy [22]. According to Lee et al. [23], symptomatic strictures were prevalent in 21% of individuals undergoing treatment for head and neck cancer using concurrent chemoradiation therapy. Female gender, initial tumors located in the hypopharynx, and twice-daily radiation therapy were important risk factors identified in the study. While the frequency of strictures associated with concurrent chemotherapy has not been much studied, these treatments are known to cause enhanced mucosal toxicity, these risk factors were consistent with our study.

Repeat dilatations are often necessary for postoperative and postradiotherapy strictures, and combining both these treatment approaches increases the number of dilatations per stricture. While people may only require one dilation and then have no symptoms, the majority require repeated dilations and are enrolled in dilation programs. The recurrence of the lesion is explained by transmural fibrosis and circular involvement due to radiation, peri-anastomotic stricture, or both.

According to recent statistics, 43% of patients had radiation strictures that did not respond to dilatation therapy [24]. Prior chemotherapy and radiation therapy may affect the stricture's refractoriness and dilatation response. Prior chemotherapy inhibits fibroblast proliferation, which may lessen the stricture's refractoriness, but more dilations were needed after earlier radiotherapy to reach 15 mm [15]. Radiation therapy was administered to these patients for a variety of malignancies, the most common of which were oesophageal cancer (19%) and head and neck cancer (51%). Scolapio et al. [25] discovered that proximity to non-esophageal cancer constituted a separate risk factor for refractory strictures. Proximal strictures were also found to be a risk factor for multiple dilatations. Thus, it is likely that radiation strictures following definitive chemoradiation for esophageal cancer have a better response to dilatation therapy than proximal strictures resulting from radiation for head and neck cancer.

We notice three major adverse events in the current investigation: three patients developed a leak that was treated conservatively with nil per oral and antibiotics for 72 hours. No patient had free perforation requiring surgery. Mild hemorrhage and retrosternal discomfort are examples of minor adverse events observed in a few patients and were managed conservatively.

In the current investigation, patients with refractory and recurring strictures required steroids more frequently. Kochhar and Poornachandra [26] demonstrated that steroid injections may augment the effect of endoscopic dilatation of varied etiology at various sites and should be considered in patients with recurrent and refractory strictures.

Strengths of our study include a large number of patients, The long follow-up data on the behavior of strictures as per the location and etiology and in patients with pliable stricture pediatric scope was passed across the stricture into the stomach, and a guidewire was passed through it to ensure the position of guidewire under direct vision. 

The limitation of our study was that our center is a tertiary care unit that receives mostly patients of advanced nature of the disease, so interventions in the early course of the disease have not been dealt with in our study. The tendency for many patients to modify their diet by avoiding solid foods in order to cope with swallowing difficulties is a topic that has not yet been covered for patients with long-term follow-up in the literature. These patients learn to live with some of these dietary constraints.

## Conclusions

Esophageal dilatation for strictures produces a good outcome with only minor adverse effects. However, the selection of patients is of utmost importance for achieving good results. Upper esophageal strictures require a greater number of sessions for adequate dilation compared to middle and lower. Post-radiation, post-surgical, and post-corrosive strictures are more likely to require repeated sessions for symptomatic relief. The ultimate objective for dilatation of esophageal strictures should be what is safe and acceptable to the patient, rather than a certain lumen size.
